# Efficiency of eSource Direct Data Capture in Investigator-Initiated Clinical Trials in Oncology

**DOI:** 10.1007/s43441-024-00671-0

**Published:** 2024-07-02

**Authors:** Hiroko Yaegashi, Yukikazu Hayashi, Makoto Takeda, Shih-Wei Chiu, Haruhiko Nakayama, Hiroyuki Ito, Atsushi Takano, Masahiro Tsuboi, Koji Teramoto, Hiroyuki Suzuki, Tatsuya Kato, Hiroshi Yasui, Fumitaka Nagamura, Yataro Daigo, Takuhiro Yamaguchi

**Affiliations:** 1 Division of Biostatistics, Tohoku University Graduate School of Medicine, 1-1, Seiryo-machi, Aoba-ku, Sendai, 980-8575 Japan; 2 A2 Healthcare Corporation, 1-4-12, Utsubohommachi, Nishi-Ku, Osaka, Japan; 3 Clinical Research Data Center, Tohoku University Hospital, Miyagi, Japan; 4 Kanagawa Prefectural Hospital Organization, Yokohama, Japan; 5 Kanagawa Cancer Center, Yokohama, Japan; 6 The Institute of Medical Science, The University of Tokyo, Tokyo, Japan; 7 National Cancer Center Hospital East, Kashiwa, Japan; 8 Shiga University of Medical Science, Otsu, Japan; 9 Fukushima Medical University, Fukushima, Japan; 10 Hokkaido University Hospital, Sapporo, Japan

**Keywords:** eSource, Direct data capture, Electronic data capture, Clinical trial, Greater efficiency

## Abstract

**Background:**

Clinical trials have become larger and more complex. Thus, eSource should be used to enhance efficiency. This study aimed to evaluate the impact of the multisite implementation of eSource direct data capture (DDC), which we define as eCRFs for direct data entry in this study, on efficiency by analyzing data from a single investigator-initiated clinical trial in oncology.

**Methods:**

Operational data associated with the targeted study conducted in Japan was used to analyze time from data occurrence to data entry and data finalization, and number of visits to the site and time spent at the site by clinical research associates (CRAs). Additionally, simulations were performed on the change in hours at the clinical sites during the implementation of eSource DDC.

**Results:**

No difference in time from data occurrence to data entry was observed between the DDC and the transcribed data fields. However, the DDC fields could be finalized 4 days earlier than the non-DDC fields. Additionally, although no difference was observed in the number of visits for source data verification (SDV) by CRAs, a comparison among sites that introduced eSource DDC and those that did not showed that the time spent at the site for SDV was reduced. Furthermore, the simulation results indicated that even a small amount of data to be collected or a small percentage of DDC-capable items may lead to greater efficiency when the number of subjects per site is significant.

**Conclusions:**

The implementation of eSource DDC may enhance efficiency depending on the study framework and type and number of items to be collected.

## Introduction

In November 2016, the ICH E6(R2) guidelines were revised, and the increase in scale, complexity, and cost of clinical trials was considered during the revision [1]. Getz et al. reported an increase of approximately 52% in the number of unique procedures to be followed in Phases I–III and an increase of 48% in the cost per patient visit in clinical studies conducted in 2011–2015 compared with those conducted in 2001–2005 [2]. Thus, the use of eSource has been explored as a methodology to achieve more efficient clinical trials.

eSource can be classified into several categories based on their characteristics. TransCelerate BioPharma Inc.’s eSource initiative classified eSource into following four categories: case report form (Non-CRF), Devices and Apps, electronic health record (EHR), and direct data capture (DDC) [3, 4]. DDC using electronic case report forms (eCRFs) as source data is one of these four categories. “eSource DDC” has different definitions in the literature. However, in this study, we defined eSource DDC as eCRFs for direct data entry. In conventional clinical trials, source data are generally kept in medical records, worksheets, or trial information entry fields created on the electronic health record. Then, the data are transcribed into eCRFs. Therefore, creating and managing worksheets and transcribing source data to electronic data capture (EDC) requires considerable time and effort, and this process can lead to missing or incorrect transcription. Thus, source data verification (SDV) is conducted to check the source data against the eCRFs. Although the concept of risk-based approach (RBA), which aims to optimize clinical trial quality by considering risks to the subject’s safety and data quality and implementing effective monitoring strategies, is becoming more widely accepted these days, and SDV tends to be risk-based, it is still one of the most resource-intensive tasks. eSource DDC is a method in which data are first recorded in eCRFs as they occur, so the eCRF data become the source data. This has the advantage of reducing the need for subsequent data transcription and SDV to verify the transcribed and source data, and consequently, the time to data finalization could be reduced. Furthermore, the qualification opinion issued by the European Medicines Agency in November 2018 [5] regarding eSource DDC stated that replacing study-specific data currently recorded on paper worksheets with eSource DDC is expected to reduce transcription work and improve data quality.

However, cases of eSource DDC implementation are still few in Japan. A survey conducted by the Japan Pharmaceutical Manufacturers Association among 50 Japanese pharmaceutical companies in September 2022 [6] revealed that only 9 out of the 50 companies have implemented eSource DDC. Looking at the characteristics of the 11 trials conducted by the 9 companies, 6 trials (55%) were healthy adult studies. Additionally, 33 out of 50 companies in the same survey answered that they have no specific plans to use eSource DDC; moreover, the most common reason for not implementing eSource DDC was “waiting for the industry to accumulate more case studies” (23/33 companies, 70%). Specifically, in case of patient trials, types of data collected and scale of participating sites may differ depending on the disease area; furthermore, the introduction of eSource DDC may have different effects on the time to access data and the hours needed for monitoring and operation of sites. However, no studies have quantitatively summarized the effect of eSource DDC on the time and hours of patient trials that have actually implemented eSource DDC.

Thus, this study aimed to evaluate the impact of the multisite implementation of eSource DDC on efficiency by analyzing data from a single investigator-initiated clinical trial in oncology conducted between 2014 and 2022.

## Materials and Methods

### Data Sources and Data Acquisition Methods

Operational data associated with a single, Phase II, multicenter, investigator-initiated clinical trial conducted in Japan was used for this analysis. Table 1 shows the source clinical trial of our eSource DDC research. The target therapeutic area was cancer, and the participating sites were either university hospitals or cancer-specialized hospitals, which had more than 100 beds and multiple departments. In general, clinical trials in which eSource DDC can be more easily implemented are characterized as Phase I trials in healthy adults, where there is less need to input information into medical records or share information with other departments. Additionally, Phase II or later trials are considered suitable when the participating sites are small clinics or hospitals with a single department, making it easier to share information within the hospital. Current ICH-E6 states that the protocol and other referenced documents should include “The identification of any data to be recorded directly on the CRFs (i.e., no prior written or electronic record of data), and to be considered to be source data” [1]. In the investigator-initiated clinical trials covered herein, each site created a source data identification list (SDIL) to identify the source data to be entered into the EDC for each eCRF item. The data used included raw data output from EDC, database structure specifications (DSS), SDIL, audit trails output from EDC, and monitoring reports. The information necessary to perform the analysis was extracted from these data. As described above, because this trial was conducted by entering source data directly into the EDC, the audit trail of the EDC includes the audit trail of the source data. This study was reviewed and approved by the Ethics Committee of Tohoku University Graduate School of Medicine.

**Table 1:** Overview of the clinical trial that implemented eSource DDC

Country	Japan
Disease area	Oncology
Phase	II
Primary endpoint	Recurrence-free survival time
Number of subjects^a^	129
Observational period	100 weeks (57 visits)
Number of sites^b^	6

^a^Number of subjects includes screening failure

^b^Five sites implemented DDC, and one site conducted the trial using conventional methods of the six participating sites

### Varying Collection Items to be Source Data at Each Site

The SDIL defines the fields where the eCRF is the source data (DDC) and those with other source data (non-DDC). In non-DDC, work hours are required to create the source documents and transcribe them to the EDC. Additionally, SDV is important for checking the consistency of these data. Therefore, all fields defined in the DSS were classified as DDC, Non-DDC and DDC/Non-DDC based on the SIDL definition by site. Audit trails were used to identify the distribution of the person who had initially entered each eCRF item into EDC.

### Time from Data Occurrence to Data Entry and Finalization

The date of data occurrence was identified for each field. For example, for the field related to weight measurement, the date of data occurrence was set to the date of weight measurement. The number of days from initial data entry to data finalization was calculated from the audit trails. Data finalization was defined as the date on which the freeze flag was applied to the final data. If the data had been modified after the freeze flag was applied, it was defined as the date on which the last freeze flag was applied The use of the freeze flag varies according to the operational policy of each clinical trial; however, in this trial, the freeze flag was applied by clinical research associates (CRAs) after the completion of data review by both the CRAs and data managers (DMs).

### Number of Visits to the Site and Time Spent at the Site by CRAs

The number of times the CRAs visited each site and the time at which the work began and ended were extracted from the monitoring reports. Visits were counted if the visit purpose included the SDV of subject data. Because the EDC data included subjects with screening failures, the number of site visits and the work time at sites by CRAs were calculated per site per subject’s visit.

### Simulation on the Impact of Change in the Clinical Trial Scale and the Percentages of DDC Fields on Site Work Hours

Kellar et al. demonstrated that challenges in implementing eSource DDC include the time required to initiate the trial and the associated costs. Additionally, site training and site resistance are also recognized as potential obstacles [4]. It is crucial to anticipate changes in site effort in advance. However, the data collected in this trial did not allow us to examine the hours required at clinical sites. Therefore, we conducted a simulation to assess the impact of changes in the clinical trial scale and the proportion of eSource DDC fields on site work hours, based on the study by Eisenstein et al. [7].

In the simulation, we assumed various combinations for the total number of data fields, the percentage of those that can be DDC, and the number of subjects. In addition to these conditions, we set the data entry speed and the additional time needed to set up the eSource DDC, compared with the time necessary for traditional study (i.e., entering source data into the medical record or paper worksheet and transcribing the data to the EDC), as fixed values under hypothetical conditions. Regarding the data entry speed, we assumed that 134 fields could be entered per hour, using the same values as in the study by Eisenstein et al. For a simplified simulation, the time required for data correction by issuing a query is not considered in this simulation. When considering the additional time required for implementing eSource DDC, it was assumed that two physicians and two CRCs would be assigned to the project. A total of 44 h was allocated for training, creating source data identification lists, conducting site user acceptance testing, and confirming the worksheet items to be implemented in the EDC (Table 2). These are hypothetical figures and may vary based on factors like the number of personnel assigned and the site’s prior experience with eSource DDC. Under these assumptions, X is the number of subjects, whereas Y is the number of hours (h) at the site. The hours Y for eSource DDC was calculated as follows: $\ \{ (\frac{{\left( {number\;of\;total\;fields - number\;of\;DDC - capable\;fields} \right)}}{{134}} + \frac{{number\;of\;total\;fields}}{{134}}) \times number\;of\;subjects\} + 44$, while that for the traditional method was calculated as $(\frac{{number\;of\;total\;fields}}{{134}} + \frac{{number\;of\;total\;fields}}{{134}})*number\;of\;subjects$. The first half of the brackets represent the time required to enter the source data outside the EDC and assume that 134 fields can be entered per hour. The second half of the brackets represents the time to enter data into the EDC.

**Table 2:** Additional resources associated with eSource DDC implementation compared with the traditional method

	Investigator (for 2 persons) (h)	CRC (for 2 persons) (h)
General education on eSource DDC implementation	5	5
Preparation for eSource DDC implementation^a^	2	20
Consideration of operational data as eSource	2	10
Total number of preparation resources	9	35

^a^Preparation for eSource implementation includes creating the source data identification list and UAT

The simulation was conducted in two steps. The first step involved fitting the number of data fields and the percentage of DDC-capable fields to match those in the actual clinical trial. These values were determined based on the results of “valuing collection items to be source data at each site” as outlined in this study. In the second step, we established eight different patterns for Simulation Sites 1–9 by varying the combinations of the number of data fields and the percentages of DDC fields. Cutoff values were determined for the number of subjects. That is, the threshold at which the time spent on the trial with eSource DDC was less than that with the traditional method was examined.

## Results

### Varying Collection Items to be Source Data at Each Site

Table 3 shows the distribution of the eCRF fields as defined in the SDIL. The percentage of fields with DDC was 61.9–84.5%, indicating variations across sites, excluding sites that did not implement eSource DDC. The total number of fields varied for each site due to its implementation in the EDC of operational data tailored to the operations of each site in this trial. Table 4 shows the percentage of initial entrants for the forms defined as DDC at all sites. Almost 100% of the initial entrants were clinical research coordinators (CRCs). Conversely, no trend could be seen from the initial entrants for the items defined as non-DDC common to each site.

**Table 3:** Percentages of DDC field and non-DDC field at each site

	Site A	Site B	Site C	Site D	Site E	Site F
DDC (%)	4033 (81.6)	2911 (61.9)	–	4036 (84.5)	3813 (79.8)	3204 (67.0)
Non-DDC (%)	906 (18.6)	1795 (38.1)	3735 (100)	731 (15.3)	966 (20.2)	1569 (32.8)
DDC/non-DDC (%)	–	–	–	12 (0.2)	–	10 (0.3)
Overall number of fields (%)	4939 (100)	4706 (100)	3735 (100)	4779 (100)	4779 (100)	4783 (100)

*DDC* number of fields where EDC data served as source data, *Non-DDC* number of fields where source data, other than EDC, were present, *DDC/non-DDC* number of fields defined as possible for both DDC and non-DDC

**Table 4:** List of forms that are “DDC” common to all sites and the percentage of roles of the person who initially entered data into the EDC

Form name	Role of initial entrant (%)
Checklist of tasks for each visit	Investigator (0.01) CRC (99.9)
Checklist for CRC (information about medication and adverse events)	Investigator (0.00) CRC (100.0)
Test performance record (screening test)	Investigator (0.00) CRC (100.0)
Test performance record (imaging test for tumor assessment)	Investigator (0.01) CRC (99.9)
Eligibility	Investigator (0.00) CRC (100.0)
Registration	Investigator (0.00) CRC (100.0)
Test performance record (vital sign)	Investigator (0.00) CRC (100.0)
Test performance record (laboratory test)	Investigator (0.00) CRC (100.0)
Test performance record (biomarker)	Investigator (0.00) CRC (100.0)
Test performance record (ECG)	Investigator (0.00) CRC (100.0)

*CRC* clinical research coordinator; for all data entered by the CRCs, the principal investigators verified them and digitally signed them

### Time from Data Occurrence to Data Entry and Finalization at Each Site

**Fig. 1:**
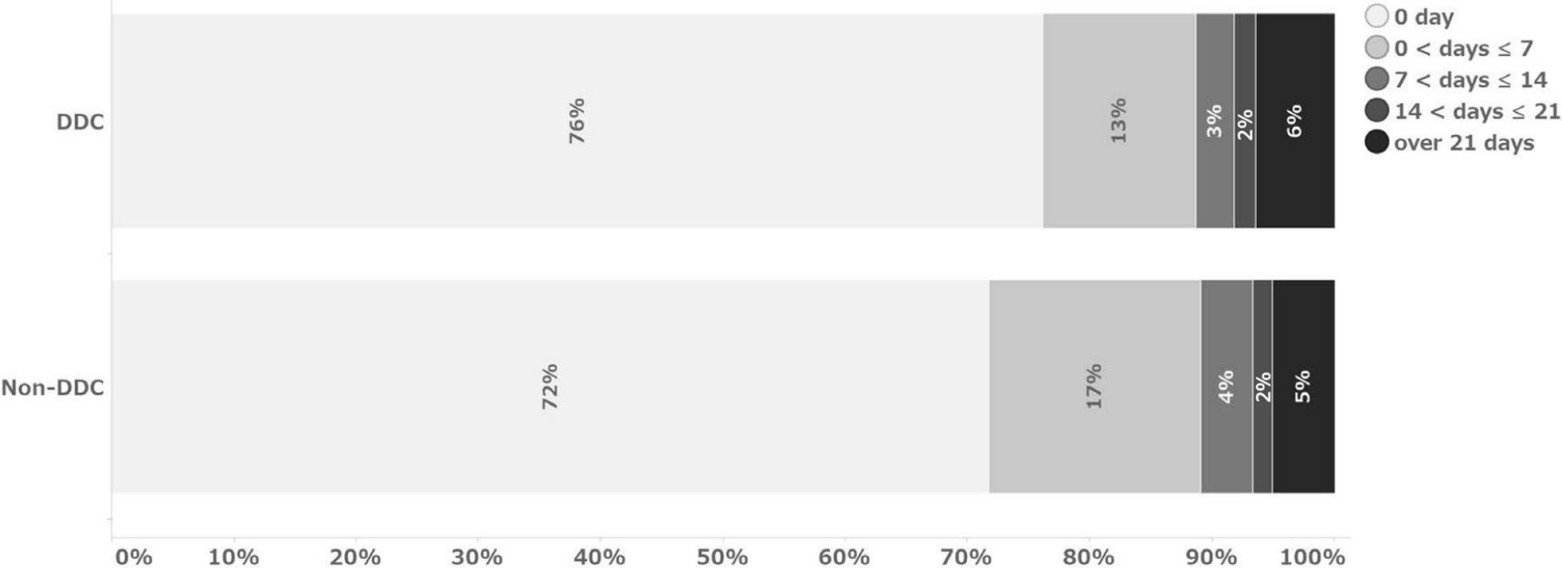
Percentage of days from data occurrence to initial data entry

**Fig. 2:**
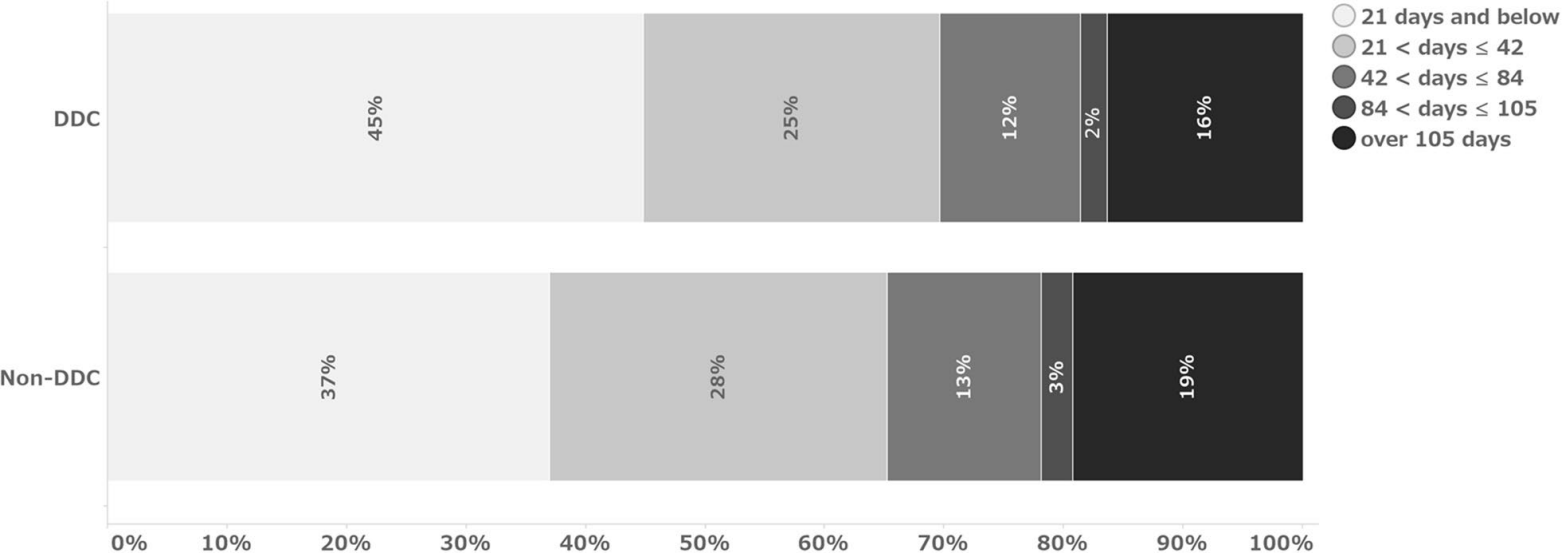
Percentage of days from data occurrence to data finalization

Figures 1 and 2 show the number of days from data occurrence to initial entry and from data occurrence to finalization, respectively. In this analysis, the median value was referred to as the representative value due to the non-normal distribution. No difference in time from data occurrence to data entry was observed between the DDC field and the transcribed data field (Table 5). The median values indicate that the data were entered in both cases on the same day of data occurrence. Regarding the number of days from data occurrence to data finalization, the DDC fields could be finalized 4 days earlier than the non-DDC fields, with a median of 24 days for the DDC fields and 28 days for the non-DDC fields.

**Table 5:** Number of days from data occurrence to data finalization

		DDC	Non-DDC
From data occurrence to initial data entry	Number of total fields entered	58,288	54,081
	Median (IQR) (days)	0 (0–0)	0 (0–1)
From initial data entry to data finalization	Number of total fields entered	58,288	54,081
	Median (IQR) (days)	22 (9–49)	27 (14–62)
From data occurrence to data finalization	Number of total fields entered	58,288	54,081
	Median (IQR) (days)	24 (11–55)	28 (16–64)

*IQR* interquartile range

### Number of Visits to the Site and Time Spent at the Site by CRAs

Table 6 shows the site information and the results of the analysis. The number of site visits by CRAs per subject per visit was 0.14 for sites with DDC and 0.14 for those with non-DDC, showing no difference. The total time spent by CRAs on site visits was 43 min at sites with DDC and 52 min at sites with non-DDC, with a difference of 9 min per subject per visit.

**Table 6:** Number of site visits by CRAs and the time spent working at sites (per subject per visit)

	Sites implemented DDC	Sites implemented non-DDC
No. of CRA visits to sites per a subject’s visit (times)	0.14	0.14
Time CRA spent at site per a subject’s visit (min)	43	52

Number of sites and subjects in each group: Sites implemented DDC (5 sites, 113 subjects), Sites implemented non-DDC (1 site, 16 subjects). Number of total visits of subjects in each group: Sites implemented DDC (2259 visits), Sites implemented non-DDC (312 visits)

*CRA* clinical research associate

### Simulation on the Impact of Change in the Clinical Trial Scale and Percentages of DDC Fields on Site Work Hours

**Fig. 3:**
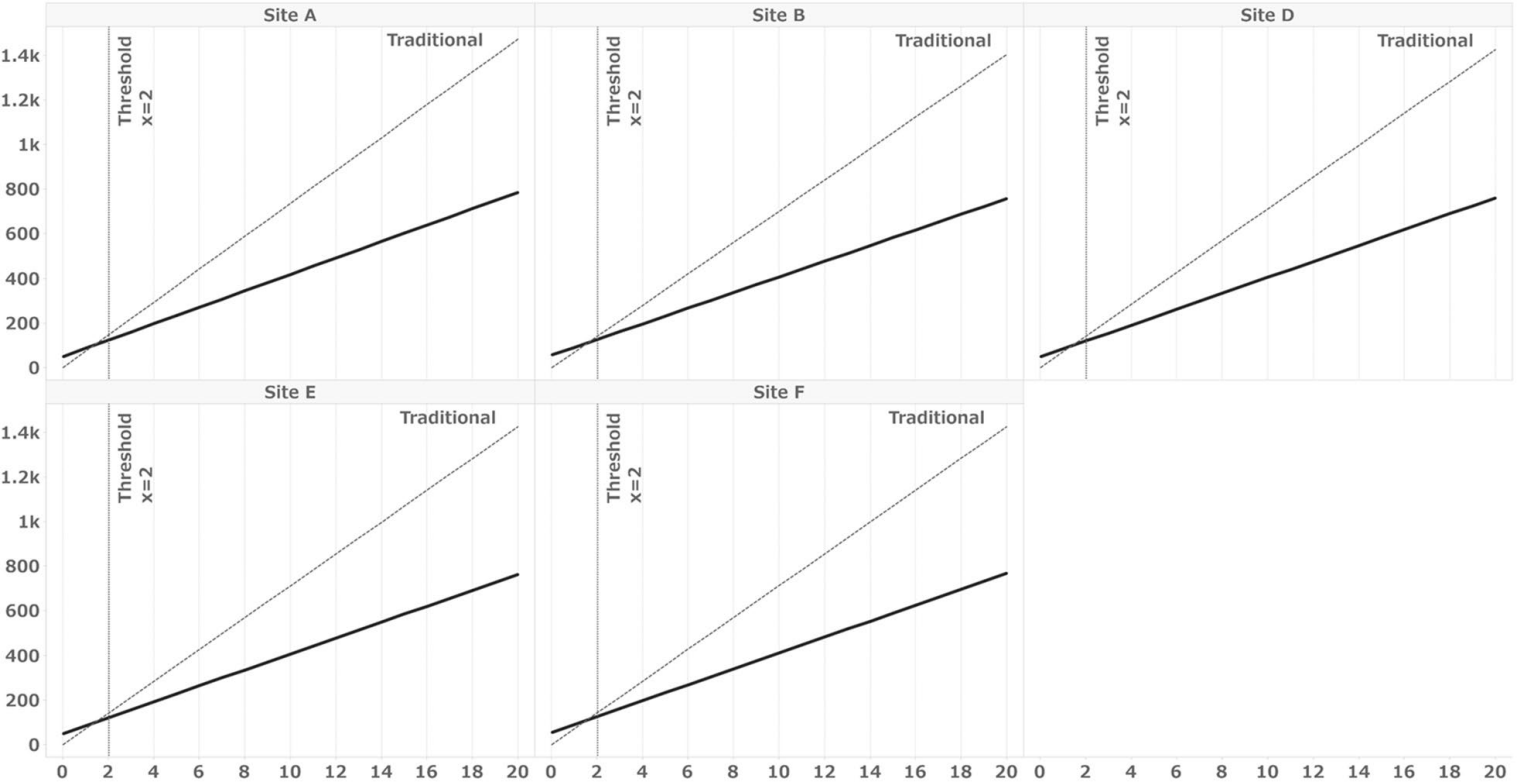
Simulation results for Sites A to F under the conditions listed in Table 3. X = number of subjects, Y = work hours, solid line: eSource DDC, broken line: traditional method. Thresholds: The minimum number of subjects for which the overall hours with eSource DDC are less than those for a clinical trial conducted using the traditional. Table 3 shows the distribution of the eCRF fields as defined in the SDIL. The number of fields included in the form defined as DDC in SIDL was used as the condition for this simulation as the number of DDC fields

**Fig. 4:**
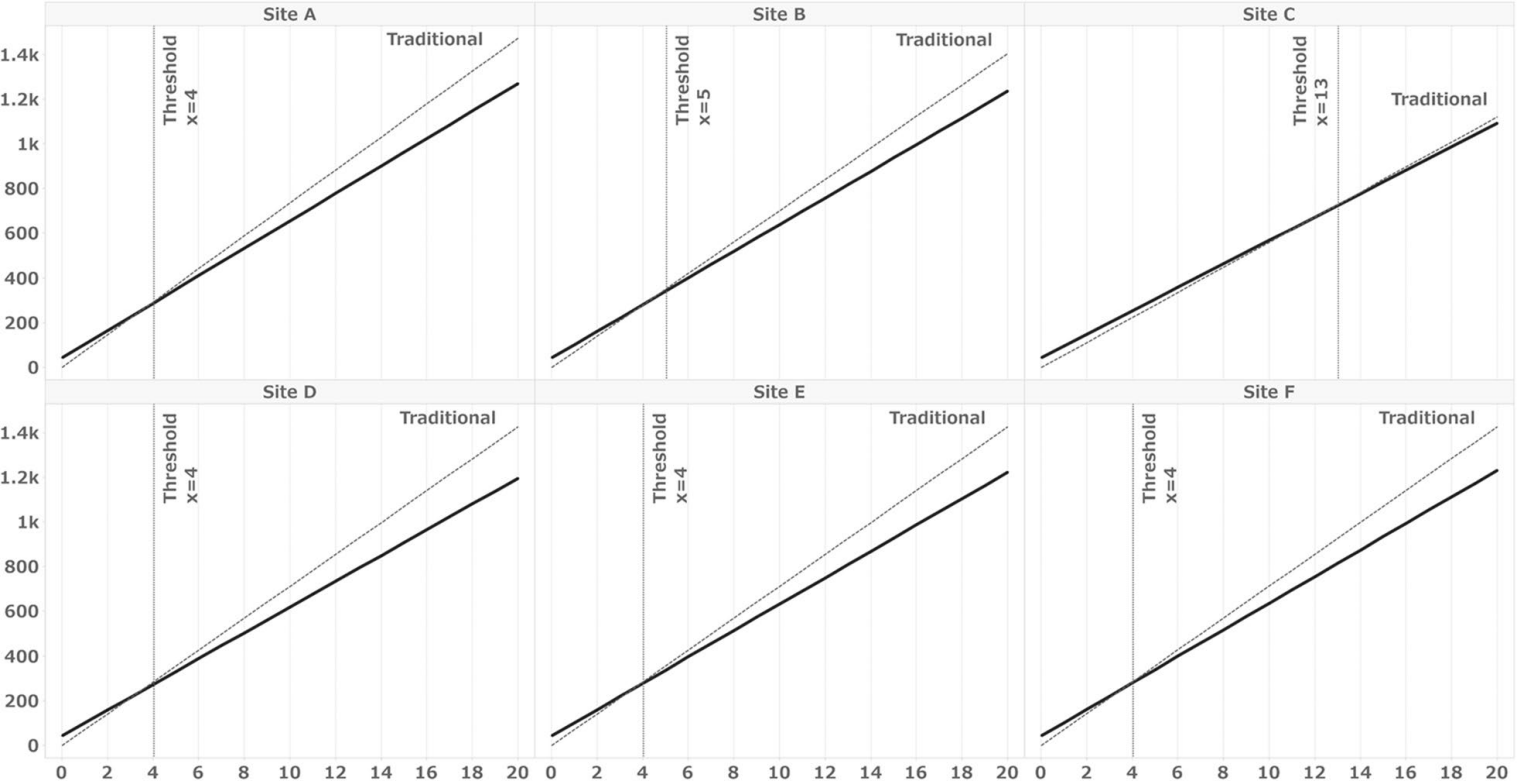
Simulation results for Sites A to F under the conditions listed in Table 4. X = number of subjects, Y = work hours, solid line: eSource DDC, broken line: traditional method. Thresholds: The minimum number of subjects for which the overall hours with eSource DDC are less than those for a clinical trial conducted using the traditional. Table 4 shows the percentage of initial entrants for the forms defined as DDC at all sites. The number of fields included in the form defined as DDC common to all facilities in Table 4 was used as the condition for this simulation as the number of DDC fields

**Fig. 5:**
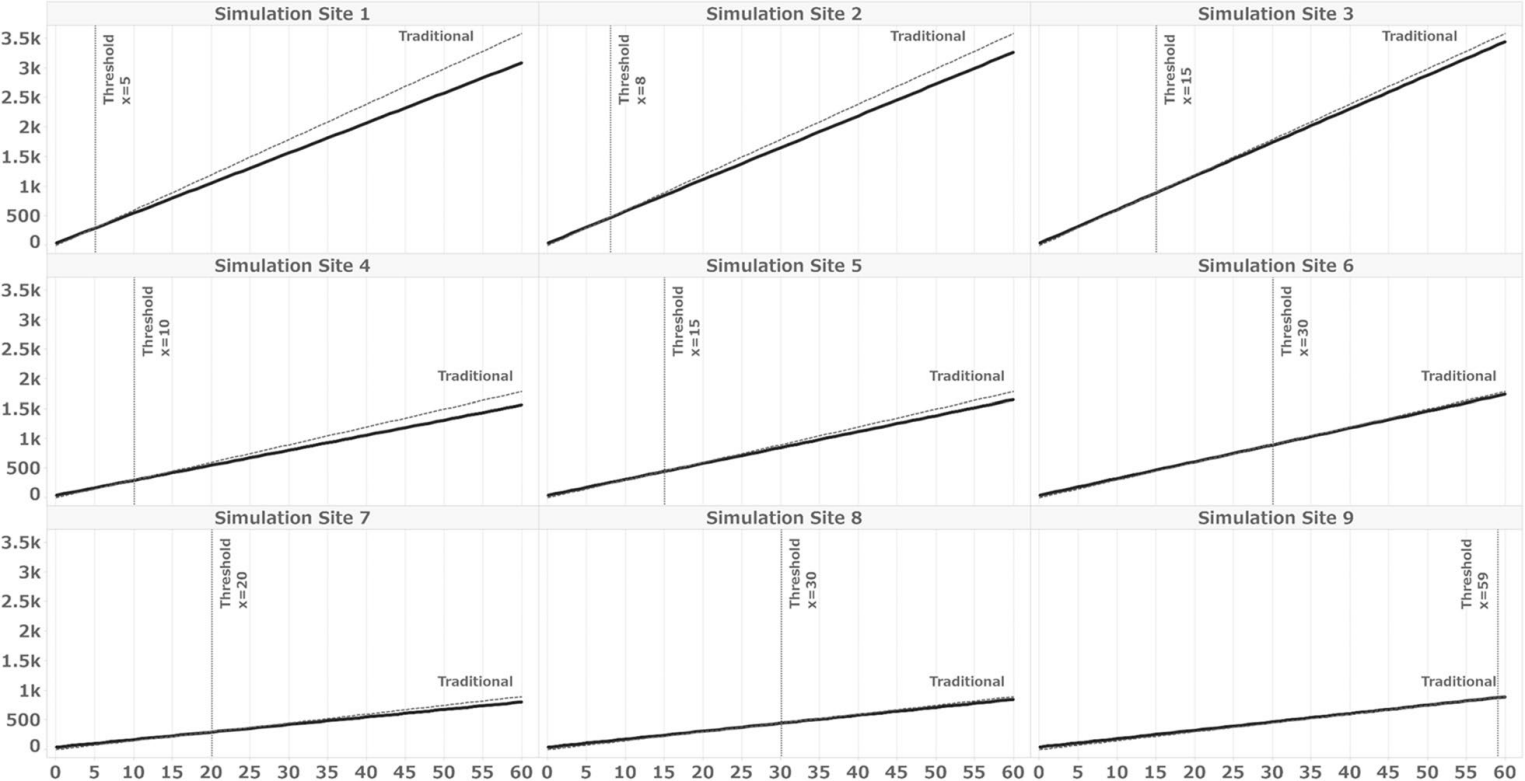
Simulation results for Sites 1–9. X = number of subjects, Y = work hours, solid line: eSource DDC, broken line: traditional method. Thresholds: The minimum number of subjects for which the overall hours with eSource DDC are less than those for a clinical trial conducted using the traditional

Table 7 displays the simulation conditions and results for this actual trial. The number of fields and DDC-capable fields were derived from the data presented in Table 3. Under these conditions, the thresholds for the minimum number of subjects required to make the overall hours with eSource DDC lower than those for a clinical trial conducted using the traditional method are illustrated in Fig. 3. The results indicate that the threshold was two subjects for all sites except Site C, which did not implement eSource DDC. Additionally, regarding the percentage of DDC-capable fields, we also simulated the percentage of fields on the form commonly defined as DDC in SDIL for all sites as DDC-capable fields, as shown in Table 4. Under these conditions, the thresholds were four subjects for Sites A, D, E, and F; five for Site B; and 13 for Site C (Fig. 4). Table 8 outlines the simulation conditions. Number of total data fields was set to 4000, 2000, and 1000, and the percentage of DDC fields to 30%, 20%, and 10%, respectively, and for a total of 9 patterns of condition combinations, assuming a smaller number of total data fields or an even smaller percentage of total data fields than those in the present trial. Figure 5 illustrates the thresholds when the number of overall fields and percentages of DDC items differ. The results demonstrate that the more DDC fields there were, the lower the minimum number of subjects that naturally became the threshold.

**Table 7:** Simulation conditions for sites A–F

	Site A	Site B	Site C	Site D	Site E	Site F
No. of data fields	4939	4706	3735	4779	4779	4783
% of data fields for DDC	81.7	61.9	0	84.5	79.8	67.0
% of the total number of fields included in the form in Table 4^a^	33.5	30.2	12.6	31.3	31.3	31.3
No. of subjects	15	64	16	14	10	10

^a^Table 4 shows the forms defined as DDC at all sites

**Table 8:** Simulation conditions for simulation sites 1–9

	Simulation condition		
Simulation site	No. of data fields	No. of data fields for DDC	Percentage of data fields for DDC (%)
1	4000	1200	30.0
2	4000	800	20.0
3	4000	400	10.0
4	2000	600	30.0
5	2000	400	20.0
6	2000	200	10.0
7	1000	300	30.0
8	1000	200	20.0
9	1000	100	10.0

## Discussion

Variations were observed among the sites participating in this trial for items for which EDC was the source data. These variations may be due to the fact that each site has different rules for operating medical records, and the examination sequence process is different. These are the factors that are difficult to change on a trial-by-trial basis. Therefore, it is necessary to acknowledge these differences as facts and plan for a reasonable operation that places less burden on the site when implementing eSource DDC. The results also suggested that the percentage of items that can be initially entered by the CRCs is an indicator of the efficiency prediction of implementing eSource DDC because if the CRCs can enter data into the EDC and use it as source data, the amount of transcription work will be reduced. Many of the items that investigators directly record require medical decisions based on multiple observations. Additionally, in many cases, it is necessary to react based on the situation. Therefore, it may be appropriate to plan the clinical trial on the assumption that those items are difficult to be DDCs.

**Fig. 6:**
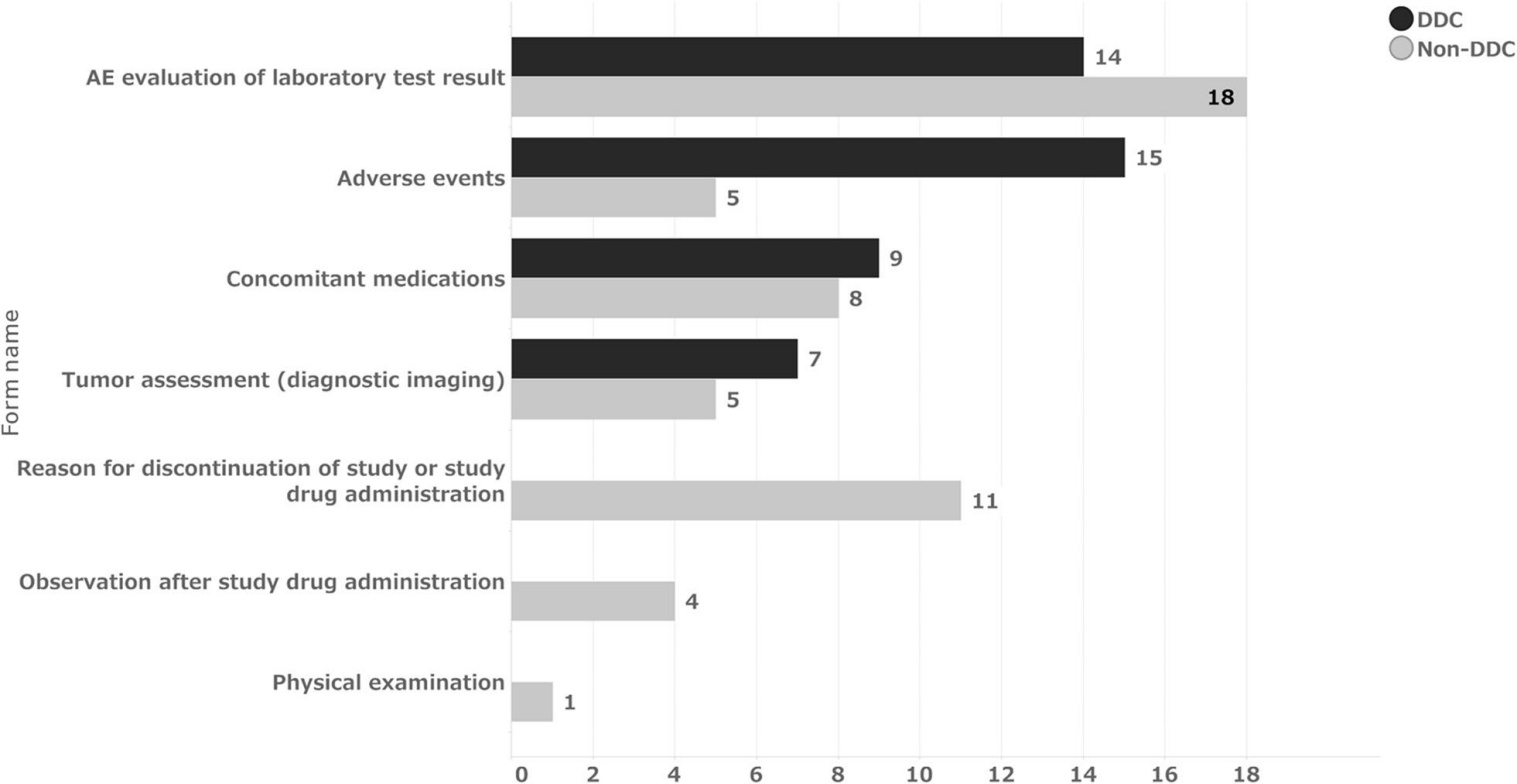
Median of days from data occurrence to initial data entry by forms. Median = 0 for all forms expect the above

The time from data occurrence to initial data entry is mainly the result of site operations. In contrast, the time from initial data entry to finalization results from operations, including SDV and data review by CRAs and DMs, in addition to site operations. Regarding the percentage of initial data entry on the day of data occurrence, 76% of DDC items and 72% of non-DDC items were entered on the same day of data occurrence, with a median of 0 days for both groups (Fig. 1). This suggests that non-DDC items; that is, data transcribed from source data recorded on other media, were also entered into the EDC in a timely manner. Thus, no difference was observed between the two groups. Figure 6 shows the distribution of the median number of days from data occurrence to initial data entry for each form. Only a few forms, such as “Tumor Assessment,” “AE Evaluation of Laboratory Test Result,” “Concomitant Medications,” and “Adverse Events,” took significantly longer to be initially entered for the DDC items. These forms were also prominent for non-DDC items. The median time from initial entry to data finalization for DDC and non-DDC items was 22 and 27 days, respectively (Table 5). This indicates that DDC items were finalized five days earlier than non-DDC items after the initial data entry. This may be because DDC items had fewer SDVs required. Furthermore, according to DMs, implementing eSource DDC allowed DMs to review some of the operational data on the EDC and infer the operation of the site, thus avoiding unnecessary queries, such as a query to verify just to be sure of the data, which may have reduced each role’s query effort.

The study results indicate that the time from data occurrence to data finalization was shortened by a median of 4 days. When considering this period in two parts—data occurrence to initial data entry and initial data entry to data finalization—the findings indicate that the implementation of eSource DDC may have a more significant impact on operations following the initial data entry than on the time from data occurrence to the initial data entry. The interpretation of this 4-day difference depends on the development strategy and other factors.

No difference in the number of visits by CRAs to sites was observed between DDC and non-DDC sites. The time spent by CRAs was 43 min per visit at sites with DDC and 52 min per visit at sites with non-DDC, with a difference of 9 min per subject per visit. This trial’s total number of visits was 57 if all trial procedures were completed. This means that the CRA time spent at the site was reduced by 8.6 h per subject. One reason for the reduced time spent may be that the items to be confirmed during the site visit could be identified beforehand.

This simulation served as a supplementary analysis to assess the impact of implementing eSource DDC on site work hours. When applying the results obtained from the SDIL of this clinical trial to the simulation conditions of the actual sites, we found that the threshold number of subjects for which eSource DDC required less time than the traditional method was 2 for all sites except Site C. However, as mentioned above, some fields defined as DDC in the SDIL still required data entry into medical records. Consequently, the percentage of DDC fields might be smaller than that defined in the SDIL. To account for this variation, we calculated the percentage of fields commonly defined as DDC for all sites in Table 4 and simulated scenarios with limited percentages of DDC fields. The results ranged from 4 to 13 subjects, representing those who completed all 57 visits. When considering the number of subjects who completed 57 visits at each site, as shown in Table 7, it is possible that the implementation of eSource DDC did not reduce work hours for Site E and Site F. The results from Simulation Sites 1–8, which represent entirely hypothetical conditions, reveal an inverse relationship between the number of DDC-capable fields and threshold subjects. These findings indicate that even a small amount of data to be collected and a limited percentage of DDC-capable items may lead to greater efficiency when the number of subjects per site is large.

Thus, to the best our knowledge, this is the first study to quantify the effect on time and work hours in a multicenter study of patients with eSource DDC. Moreover, this study was based on a single trial, and there was only one site with non-DDC, which was analyzed to compare the number of site visits and time spent by monitors. Additionally, there is a limitation in terms of generalizability because the study was designed in 2019, the targeted clinical trial began in 2014, and more information needed to be collected on the background information required to extrapolate the results. However, many organizations are “waiting for the industry to accumulate more case studies” as a reason why they have no specific plans to introduce eSource DDC [6], and we believe that this study can encourage further research by introducing our case study to promote the introduction of eSource DDC into appropriate clinical trials. Moreover, the source clinical trial was initiated in 2014, when the concept and precedent of eSource DDC was still even less common than it is today. Therefore, it is undeniable that there could have been a more efficient way to implement eSource DDC and perhaps resulting in less homogeneity of eSource DDC across the study sites. However, even under such circumstances, work hours required for monitoring and simulation showed that the site reduced its work hours. We are not suggesting that eSource DDC should be implemented in all clinical studies. The most important thing is the ability to collect data to achieve the study objectives while ensuring the safety of the subjects. However, eSource DDC can be partially implemented and may lead to greater efficiency even if only some of the items are DDC as simulated.

## Limitations

The result was based on a single study with 6 sites, and there is a limitation in generalizability. Thus, we believe that a cluster-randomized study in which sites are randomized to DDC and non-DDC would allow for a more non-biased analysis. Although some EDC data were defined in advance as source data in the SDIL, some data were transcribed after other source data were recorded, and the analysis results could not take these into account. Additionally, the EDC setup and off-site monitoring have yet to be considered in this result. Thus, careful consideration should be given to determine whether the implementation of DDC will improve efficiency throughout the entire clinical trial. Moreover, this trial was initiated in 2014, i.e., prior to the revision of ICH E6(R2); consequently, the operation was not considered from the perspective of RBA. Higher efficiency could have been anticipated if it had been combined with RBA. Further research is required to investigate this assumption. Additionally, multiple trial data with actual site’s work hours using this algorithm are needed to perform validation of the algorithm used in this study.

## Conclusions

This study proposed a case in which eSource DDC was implemented in a late-phase, multicenter, investigator-initiated clinical trial in oncology, which is considered difficult to implement in Japan. The implementation of eSource DDC may enhance efficiency, depending on the study framework and the type and number of items to be collected.

## Data Availability

The datasets analyzed during the current study are available from the corresponding author on reasonable request.
